# *Cecropia angustifolia* Pentacyclic Triterpene Acids and Sacha Inchi Oil Improve Carbohydrate Metabolism and Inflammation in Prediabetic Mice

**DOI:** 10.1155/adpp/4687213

**Published:** 2025-08-24

**Authors:** Johanna Valentina Lopez-Cortes, Sergio Acin-Martinez, Guillermo Montoya, Norman Balcazar

**Affiliations:** ^1^Facultad Barberi de Ingeniería, Diseño y Ciencias Aplicadas, Departamento de Ciencias Farmacéuticas, Biomédicas y Veterinarias, Universidad Icesi, Valle Del Cauca, Cali 760031, Colombia; ^2^Facultad de Medicina, Universidad de Antioquia, Antioquia, Medellín 050010, Colombia; ^3^BioInc., Center for Biomass Valorization Research, Universidad Icesi, Valle Del Cauca, Cali 760031, Colombia

**Keywords:** *Cecropia angustifolia*, glucose tolerance, high-fat diet, insulin resistance, pentacyclic triterpene acid (PTA), type 2 diabetes mellitus (T2DM)

## Abstract

Type 2 diabetes mellitus is closely linked with obesity and associated metabolic dysfunctions, including insulin resistance, dyslipidemia, and chronic inflammation. Pentacyclic triterpene acids (PTAs) derived from *Cecropia angustifolia* are promising bioactive compounds that may help mitigate these disorders. This study investigated the effects of a PTA-rich fraction on metabolic disruptions in cellular and diet-induced obesity mouse models. Prediabetic C57BL/6J mice fed on high-fat diet (HFD) for 8 weeks exhibited typical metabolic abnormalities, such as increased body weight, glucose intolerance, hyperinsulinemia, and dyslipidemia, which served as a baseline for assessing PTA efficacy. 8-week treatment with *C. angustifolia* PTAs showed significant improvement in glucose metabolism, enhancing insulin sensitivity and glucose tolerance and reducing plasma insulin levels. Although PTAs did not alter body weight or lipid profiles in HFD-fed mice in a sacha inchi oil (SIO) vehicle, they effectively prevented further weight gain, especially with intraperitoneal administration. Interestingly, we found that SIO, used as PTA solubilizer, yielded similar hypoglycemic and anti-inflammatory outcomes, and coadministration did not yield additive or synergistic effects. Furthermore, both PTAs and SIO demonstrated in vitro anti-inflammatory activity by downregulating proinflammatory gene expression, and no adverse liver, kidney, and pancreas toxicity was observed. In conclusion, PTAs from *Cecropia angustifolia* and SIO present potential as nontoxic, bioactive agents for modulating carbohydrate metabolism and inflammation in obesity-related conditions, although further studies are warranted to optimize dosing and investigate SIO's standalone therapeutic potential.

## 1. Introduction

Type 2 diabetes mellitus (T2DM) represents a critical and expanding global health issue, characterized by chronic dysregulation of lipids and carbohydrate metabolism. This metabolic disorder is primarily driven by chronic low-grade inflammation, insulin resistance, and β-cell dysfunction, collectively leading to sustained hyperglycemia and progressive metabolic decline [1]. The prevalence of T2DM is closely linked to the rise in obesity, especially central adiposity, as an increased body mass index (BMI) in the abdominal area constitutes a substantial risk factor for the development of insulin resistance and subsequent T2DM [2]. Although lifestyle modification such as adherence to a balanced diet, regular physical activity, and weight management is the most effective strategy to mitigate the progression of T2DM, achieving sustainable change remains challenging [2, 3]. Socioeconomic factors, urbanized lifestyles, limited access to nutritious foods, and low physical activity levels contribute significantly to the rise in T2DM. Consequently, pharmacological interventions and alternative therapeutic approaches have become vital in managing this disease. Among these alternatives, dietary supplements derived from plant sources offer a promising adjunct to conventional treatment, given their high tolerability and potential to mitigate disease progression with fewer side effects.

Plants have been a cornerstone of traditional medicine, valued for their diverse phytotherapeutic compounds. In recent decades, plant-derived nutraceuticals have garnered significant attention for their therapeutic potential across various disease states. Notably, pentacyclic triterpene acids (PTAs) have demonstrated considerable potential in managing metabolic disorders, including T2DM. PTAs are a class of terpenes composed of six isoprene units usually comprising five, six-membered rings. These triterpenoids are naturally occurring compounds found in fruits such as apples, pears, and olives, as well as in medicinal plants like basil and trees such as eucalyptus and *Cecropia* genus [4–11].

Extensive research has substantiated the beneficial effects of PTAs on various aspects of metabolic health through different mechanisms. For instance, Gutiérrez et al. [12] observed that serjanic acid administration resulted in lower cholesterol and triglyceride (TG) levels, increased serum insulin concentrations, reduced fasting blood glucose, and improved glucose tolerance and insulin sensitivity. Furthermore, ursolic and oleanolic acids derived from *Bouvardia terniflora* have shown glucose-lowering effects in both normal and diabetic murine models [13].

However, despite their therapeutic promise, PTAs face significant pharmacokinetic limitations. Their low water solubility and high lipophilicity hinder gastrointestinal absorption, resulting in poor bioavailability. Jiang et al. reported a bioavailability of just 2.3% for 23-hydroxybetulinic acid following intragastric administration in mice, while Jeong et al. found that oleanolic acid exhibited only 0.7% bioavailability in rats due to similar absorption challenges [14–16]. To address these barriers, pharmaceutical formulation techniques involving biocompatible matrices, such as encapsulation in nanoparticles, have been employed to enhance dissolution and absorption properties of PTAs. For instance, Ge et al. [17] reported that ursolic acid nanoparticles formulated with D-α-tocopheryl polyethylene glycol succinate achieved a 27.5-fold increase in bioavailability compared to the free compound. Likewise, a pharmacokinetic study with *Cecropia angustifolia* employing sacha inchi oil (SIO) demonstrated enhanced PTA absorption, improving bioavailability to 9.2% [18].

The objective of this study is to evaluate the safety and efficacy of a chemically standardized fraction of PTAs from *Cecropia angustifolia* in a SIO vehicle. Given the promising metabolic effects observed in earlier studies, this research aims to investigate the impact of these triterpenes on inflammation, obesity progression, insulin resistance, and overall metabolic health in a prediabetic mouse model. By exploring oral and intraperitoneal (IP) administration routes and the incorporation of PTAs into SIO, this study seeks to identify optimal conditions that maximize the therapeutic potential of PTAs from *C. angustifolia*, providing insight into novel strategies for managing T2DM.

## 2. Materials and Methods

### 2.1. Herbal Material and Triterpene Fraction Preparation

The roots of *Cecropia angustifolia* were collected in Pance, Valle del Cauca, at an altitude of 1771 m in July 2021, with the following geodesic location Y 3°20′12.0″ N |X 76°38′41.6″ W. The plant material was compared against the OD2126 voucher stored at the university herbarium. Access to genetic resources agreement number 180 between Universidad Icesi and the Colombian Ministry of Environment (Otrosi No. 10) was awarded on May 7 of 2018. All procedures are in accordance with the Nagoya Protocol.

The herbal material was dried in a ventilated oven for three consecutive days at 40°C and then shredded using a hammer mill. Extraction was carried out using a solid-liquid extraction semipilot plant with ethyl acetate (EA) and dichloromethane (DCM) in the same proportion at a 1:5 herbal material/solvent ratio. The mixture was stirred at 90 rpm at room temperature for 7 days, monitoring by thin-layer chromatography (TLC) until the absence of PTs. Vacuum chromatography with EA under normal phase conditions was used to defat the extract and recover the fraction with a high PT content.

### 2.2. Total Pentacyclic Triterpene Content and Profile Confirmation

**Figure 1:**
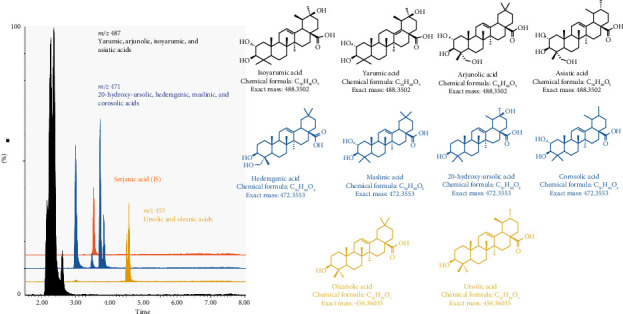
UPLC-ESI-MS in negative SIM mode acquisition in the quantitation of total triterpene content giving a controlled chemical profile. Serjanic acid was used as internal standard (a). Chemical structures described in the PTA chemical fraction (b).

*Cecropia angustifolia* is a native plant from central and northern South America. Several species of Cecropia plants have been widely used in Latin American traditional medicine to treat metabolic diseases and reported to contain abundant PT. The production of serjanic acid previously reported by our laboratory in *Cecropia telenitida* enabled its use as an internal standard [M–H]^−^*m/z* 499 to monitor quantitative expression since this triterpene is not produced by *Cecropia angustifolia* or is below the quantification threshold of our system (Figure 1a). To simplify analysis, and given the presence of multiple isobaric triterpenes in the fraction, we focused on characteristic mass-to-charge ratios: [M–H]^−^*m/z* 487, representing oxidized molecules such as yarumic, isoyarumic, arjunolic, and asiatic acids; [M–H]^−^*m/z* 471, corresponding to hederagenic, 20-hydroxy-ursolic, maslinic, and corosolic acids; and [M–H]^−^*m/z* 455, representing their less oxidized precursors, oleanolic and ursolic acids, derived from oleanyl and ursanyl cations (Figure 1b). Quantitative analysis was performed using UPLC-SQD2 mass spectrometry (Waters®). Chromatographic conditions were established previously defining a method for multiple metabolite separation. The chromatographic separation of triterpene acids was carried out on Acquity UPLC® BEH C18 (100 × 2.1 mm, 1.7 μm) and the column was kept at 35°C. The mobile phase was Mili-Q water with 5 mM of ammonium acetate (A) and methanol hypergrade (B) at constant flow rate of 0.3 mL min^−1^ with a gradient elution of 0 min at 30% A, 1 min at 20% A, 1.50 min 15% A, 3 min 5% A, 4.50 min 2% A, 6.00 min 2% A, 10 min 30% A.

The mass spectrometer uses an electrospray ionization (ESI) source, kept at 500°C and acquired in negative mode. Nitrogen was used as nebulizer and curtain gas. The optimal values for MS parameters were capillary voltage of 3.50 kV, cone voltage of 80 V, desolvation temperature of 500°C, and desolvation gas source flux of 900 L/hr. The analyzer was operated in single ion recording (SIR) mode, which monitored [M-H]^−^ ions at *m/z* of 487, 455 and 471, and serjanic acid as the IS at *m/z* 499. Masslynx MS software was used for instrument control, data acquisition, and data processing.

### 2.3. Cell Cultures

J774A.1 (TIB-67™) mouse macrophage cells and 3T3-L1 (CL-173™) mouse fibroblast cells were purchased from ATCC (Manassas, VA, USA). Cells were cultured in Dulbecco's Modified Eagle's Medium (DMEM) with 10% fetal bovine serum (FBS), 2 mM glutamine, and 1% penicillin/streptomycin (Sigma-Aldrich, St. Louis, MO, USA) at 37°C and 5% CO_2_. J774.A1, and 3T3-L1 cells were cultured with 25 mM glucose (growth medium—GM).

### 2.4. Macrophage Cell Culture and Activation

J774A.1 cells were cultured in GM. At 80% confluence, J774A.1 cells were incubated in GM containing 100 ng/mL lipopolysaccharide (LPS) and 20 ng/mL interferon gamma (IFN-γ) (activation medium—AM) for 24 h. Treatments were added during the last 6 h. MTT cell proliferation was performed, and RNA was collected to evaluate inflammation related gene transcription.

### 2.5. Adipocyte Cell Culture and Differentiation

3T3-L1 preadipocytes were cultured in GM. Differentiation was induced 2 days post-confluence by adding GM containing 0.5 mM 3-isobutyl-1-methylxanthine (IBMX), 0.25 μM dexamethasone, 2 μM rosiglitazone, and 1 μg/mL insulin. After 2 days of incubation, the medium was replaced with GM containing 1 μg/mL insulin. Two days later, the medium was replaced by GM and incubated for another 5 days with the different treatments (replacing it every 2 days). MTT cell proliferation was performed. Cells were stained to evaluate intracellular TG accumulation.

### 2.6. Cell Viability and Biological Activity Test

To evaluate cell viability, 3T3-L1 and J774A.1 cells were seeded on 96 multiwell plates at X cell/well and cultured according to the protocols. They were treated at different concentrations of PTAs, and MTT Cell Viability Assay Kit (Sigma-Aldrich, St. Louis, Mo, USA) was used. MTT was added to the cells for 2 h. After that time, formazan crystals were dissolved by adding DMSO. Absorbance was measured at 570 nm in a Varioskan™ LUX microplate reader (Thermo Fisher Scientific, Waltham, MA, USA). To evaluate the biological activity of 3T3-cells, Oil Red O staining was performed to determine the intracellular accumulation of TG. 3T3-L1 cells were differentiated and treated for 5 days. Then, cells were washed with PBS and fixed in 10% formaldehyde at room temperature for 1 h. Cells were washed with 60% isopropanol and completely dried. The fixed cells were stained with Oil Red O solution (Sigma-Aldrich, St. Louis, MO, USA) at room temperature for 30 min and washed with water. The stain of lipid droplets was extracted with 100% isopropanol and the absorbance was measured at 492 nm in a Varioskan™ LUX multimode microplate reader (Thermo Fisher Scientific, Waltham, MA, USA).

### 2.7. RNA Extraction and Real-Time PCR for J774A.1

Total RNA was extracted from J774A.1 cells with RNeasy kit (QIAGEN, Valencia, CA), and the reverse transcription reaction was performed with 500 ng total RNA, 10X RT Buffer, 25X dNTP Mix (100 mM), 10X Random Primers, MultiScribe TM Reverse Transcriptase, and RNase Inhibitor (Thermo Fisher Scientific) according to the manufacturers' instructions. Real-time quantitative PCR (qPCR) analyses were performed with 50 ng cDNA and 100 nM sense and antisense primers (Integrated DNA Technologies, Coralville, IA, USA) in a final reaction volume of 25 μL by using the Maxima SYBR Green/ROXqPCR Master mix (Thermo Fisher Scientific) and the CFX96 real-time PCR detection system (Bio-Rad). Results were normalized to the cyclophilin B (Ppib) expression level. The expression of the inflammatory marker gene interleukin 1 beta (Il1B) was evaluated, and the relative amount of the whole mRNA was calculated using the comparative or ^ΔΔ^Ct method. The sequence-specific oligonucleotide primers are Il1B F: 5′-AGAGCTTCAGGCAGGCAGTAT-3′, R: 5′-GAAGGTGCTCATGTCCTCATC-3'; Ppib F: 5′-GGAGATGGCACAGGAGGAA-3′, R: 5′-GTAGTGCTTCAGCTTGAAGTTCTCAT-3´. Annealing temperature: 60°C.

### 2.8. Biomodels, Experimental Design, and Dietary Treatment

The Institutional Animal Care and Use Committee of the Universidad de Antioquia approved all animal studies. C57BL6/J male mice (Charles Rivers Laboratories, Wilmington, MA, USA) over 4 weeks old were used. Mice were housed at 22 ± 2°C with a 12:12 h light dark cycle with free access to food and water.

The animals were adaptively fed with a normal diet for 1 week and then were randomly divided into six groups, each with 10 animals. A control group was fed a normal diet (chow, 14% fat/54% carbohydrates/32% protein). The other 5 high-fat diet (HFD) groups were fed a HFD (42% fat/42% carbohydrates/15% proteins).

Groups III to VI were fed a HFD for 8 weeks prior to administration of treatments.

• Group I. Negative control chow, normal male mice fed a normal diet.
• Group II. Model control group fed a HFD with no treatment intake.
• Group III. PTAs in PBS intraperitoneally administrated PTAs-PBS_(IP)_, fed with HFD, and subjected to an 8-week treatment (14 doses) of PTAs (300 μL at 100 mg/kg b.w.).
• Group IV. PTAs in PBS by oral administration PTAs-PBS_(Oral)_, fed with HFD, and subjected to an 8-week treatment (14 doses) (300 μL at 100 mg/kg b.w.).
• Group V. PTAs in SIO by oral administration PTAs-SI_(Oral)_, fed with HFD, and subjected to an 8-week treatment (14 doses) (300 μL at 100 mg/kg b.w.).
• Group VI. SIO SI_(Oral)_, fed with HFD, and subjected to an 8-week treatment of SIO (300 μL).

### 2.9. Glucose and Insulin Tolerance Test (ITT) in a Diet-Induced Obesity Mouse Model

An intraperitoneal glucose tolerance test (IPGTT) and ITT were performed before and after treatments. On test days, animals fasted for 6 h. IPGTT was performed on the mice by administering a glucose load of 2.0 g/kg b.w. and ITT was performed by IP injection of human insulin 0.75 U/kg (Humulin, Eli Lilly, Indianapolis, IN, USA). Blood glucose levels were measured via tail vein blood at 0, 15, 30, 60, and 120 min after the injection using a GlucoQuick Glucometer. Zero time was measured just before glucose or insulin injection. After glucose metabolism studies, the mice were sacrificed, and blood and tissue samples were collected for further analyses.

### 2.10. Serum Biochemical Parameters

At the end of the treatments, mice were sacrificed by suffocation with CO_2_. Blood was drawn from the heart. Plasma insulin level was measured using an enzyme-linked immunosorbent assay (ELISA) kit (Mercodia AB, Uppsala, Sweden). Plasma total triacylglycerol (TAG), total cholesterol (TC), LDL, alanine aminotransferase (ALT) (11832), and aspartate aminotransferase (AST) (23531) concentrations were determined by commercial assays (Biosystems). All determinations were done following the manufacturer's recommendation, and the absorbance was measured using a VarioskanTM LUX multimode microplate reader (Thermo Fisher Scientific).

### 2.11. Histological Analysis

At the end of the treatments, mice were sacrificed, and a sample of the livers, kidneys, and pancreas was stored in neutral formaldehyde and embedded in paraffin wax. Sections (4 mm) were stained with hematoxylin and eosin and observed using a Nikon microscope. A veterinary pathologist evaluated and scored different parameters in the specimens under light microscopy. For a semiquantitative comparison of the structural changes, the abnormalities in the tissue sections were scored as follows: 1 (no damage), 2 (minor damages), 3 (between minor and moderate damage), 4 (moderate damage), 5 (between moderate and severe damage), and 6 (severe damage).

### 2.12. Statistical Analysis

Results are expressed as means ± SEM. Comparison between groups were analyzed using one-way analysis of variance (ANOVA) followed by a Dunnett post hoc test. Differences were considered as significant at *p* < 0.05. All analyses were performed with the Prism 10.4.0 (GraphPad software, Inc., La Jolla, CA, USA) statistical software.

## 3. Results

### 3.1. Effect of PTAs on J774.A1 Macrophage and 3T3-L1 Adipocyte Cell Lines

**Figure 2:**
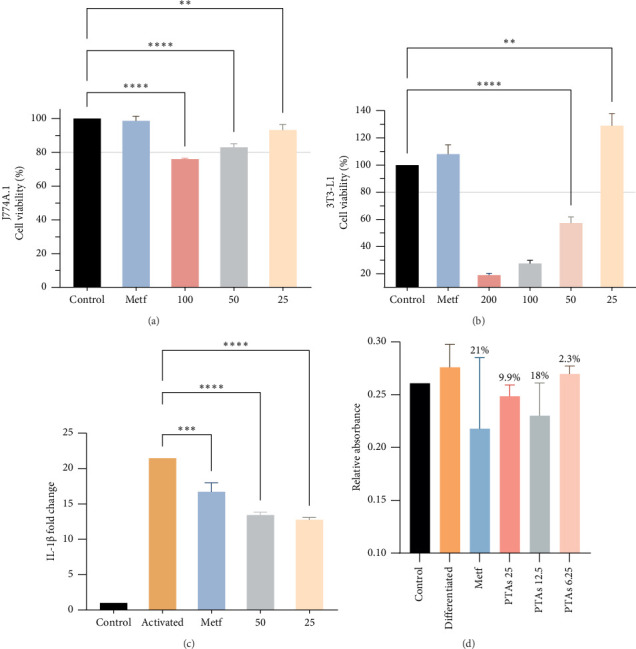
Effect of PTAs on J774.A1 and 3T3-L1 cell lines. Evaluation of cell viability in J774A.1 (a) and 3T3-L1 (b). Effect of PTAs on macrophage mRNA expression level of IL-1β (c). Effect of PTAs on 3T3-L1 adipocyte TG accumulation (d). ^∗∗^*p*  < 0.01, ^∗∗∗^*p* < 0.001, and ^∗∗∗∗^*p* < 0.0001 compared with controls.

The effect of PTAs (Figure 1) on cell viability was determined by MTT assay (Figures 2(a) and 2(b)). This technique showed that PTA concentrations superior to 50 and 25 μg/mL produced a significant reduction of more than 20% in viability of J774A.1 and 3T3-L1 cells, respectively. Thus, for the macrophage and adipocyte cell lines, PTAs' working concentrations up to 50 and 25 μg/mL were chosen, respectively.

J774A.1 macrophages were activated with LPS and INF-γ for 24 h and were treated with PTAs at 25 and 50 μg/mL during the final 6 h of activation. To determine the action of PTAs in the expression of Il1B proinflammatory gene, we performed RT-PCR. Both treatments significantly reduced the transcriptional expression of the gene mRNA in J774A.1 cells (Figure 2(c)).

The effect of the PTA treatment on 3T3-L1 adipocyte TG accumulation is shown in Figure 2(d). The lipid content of cells treated with PTAs at 25 μg/mL was reduced by 18%, a comparable effect of metformin treatment, used as positive control, that reduced fat load by 21%.

### 3.2. Effect of PTAs on Mice Body Weight and Glucose Metabolism in the Mouse Model of Diet-Induced Obesity

**Figure 3:**
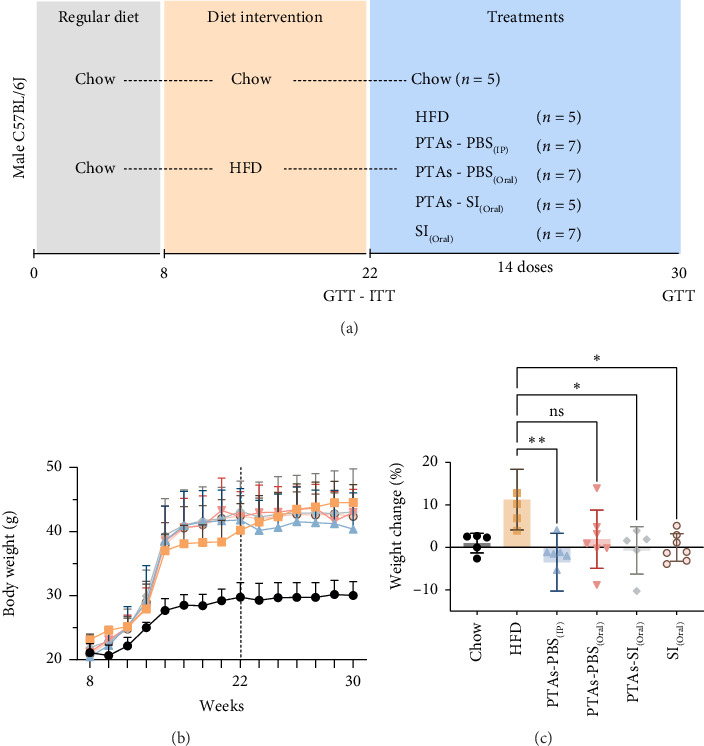
Prediabetic dietary intervention in response to high-fat diet (HFD). (a) Experimental design. Mice were randomly divided into six groups: Chow, negative control mice were fed with standard diet; HFD, positive control mice were fed with HFD and no treatment; PTAs-PBS (IP), HFD-fed mice were treated for 8 weeks with 14 intraperitoneal doses of PTAs suspended in PBS (300  μL, 100 mg/kg); PTAs-PBS (Oral), HFD-fed mice were treated orally with PTAs in PBS (300  μL, 100 mg/kg, 8 weeks); PTAs-SI (Oral), HFD-fed mice were treated orally with PTAs in sacha inchi oil (300  μL, 100 mg/kg, 8 weeks); and SI (Oral), HFD-fed mice were treated with sacha inchi oil alone. All treatments began after confirmation of insulin and glucose resistance. (b) Body weight progression between Weeks 8 and 30; treatments started at Week 22. (c) Percentage change in body weight before and after treatment. *p* < 0.05 and *p* < 0.01 vs. HFD.

C57BL/6 mice were fed a chow or HFD for 14 weeks prior to treatments (Figure 3(a)). Figures 3(b) and 3(c) show that after 14 weeks of HFD, mice gained weight compared to mice fed chow diet. No treatment reduced significantly mice body weight compared to HFD after 14 doses (Figure 3(b)); however, IP treatment of obese mice with PTAs and oral treatment with PTAs in sacha inchi and SIO significantly prevented the mice from gaining weight compared to HFD. This effect was attenuated in oral PTA treatment group (Figure 3(c)).

**Figure 4:**
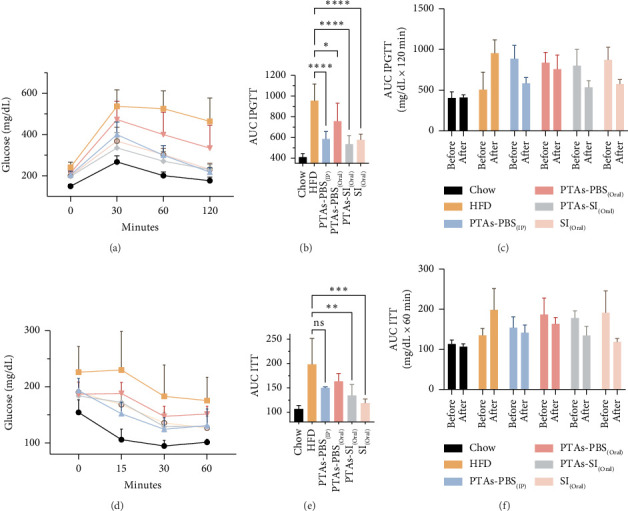
Assessment of carbohydrate metabolism. (a) Glucose tolerance test (IPGTT) and (d) insulin tolerance test (ITT) in control and treated prediabetic mice. All assays were performed after 8-week treatment. (b, e) AUC values, calculated using data obtained in (a, d). (c, f) AUC values comparing before and after treatments. ^∗^*p* < 0.05, ^∗∗^*p* < 0.01, ^∗∗∗^*p* < 0.001, and ^∗∗∗∗^*p* < 0.0001 compared with HFD.

The ITT test showed that mice fed an HFD present insulin resistance compared to mice fed the normal diet. This insulin resistance was significantly reversed when mice were treated with oral PTAs in sacha inchi and sacha inchi oil. The PTA treatments were not as effective in reversing this resistance compared to the superior effect of the SI treatments (Figures 4(d) and 4(e)). These data were confirmed by comparing ITT assays before and after treatment of each group (Figure 4(f)).

### 3.3. Effect of PTAs on Insulin and Plasma Lipid Levels in the Mouse Model of Diet-Induced Obesity

**Figure 5:**
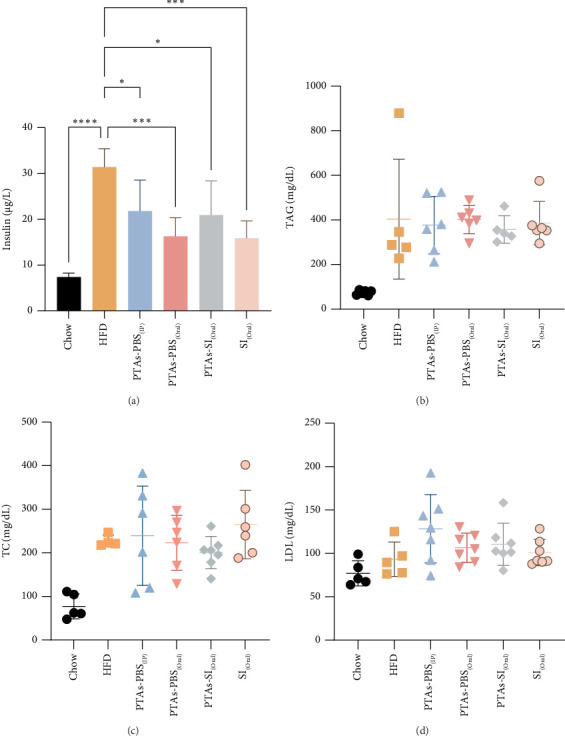
Lipid plasma and insulin evaluation. (a) Insulin plasma levels. (b) Triacylglycerides, (c) total cholesterol, and (d) LDL plasma levels of different groups. ^∗^*p* < 0.05, ^∗∗∗^*p* < 0.001, and ^∗∗∗∗^*p* < 0.0001 compared with HFD.

As shown in Figure 5, the mice fed a HFD had a significant increase of plasma insulin (Figure 5(a)). In a similar way, HFD increased TC, LDL, and TAG levels (Figures 5(b), 5(c), and 5(d)). All treatments with PTAs and sacha inchi significantly reduced the insulin levels (Figure 5(a)). However, no treatment modified lipid plasma levels (Figures 5(b), 5(c), and 5(d)).

### 3.4. Effect of PTAs on Tissue Histopathological Changes

**Figure 6:**
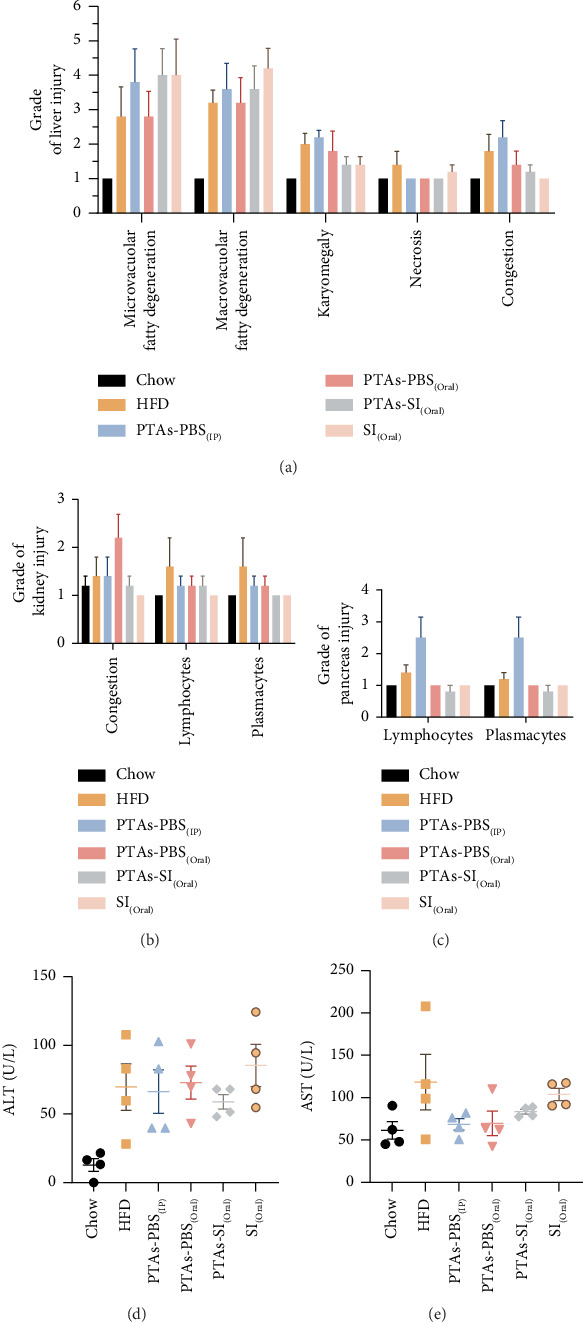
Aminotransferases and histopathological evaluation. (a, b, c) Liver, kidney, and pancreas injury level. (d, e) ALT and ALT plasma levels.

Liver histopathology confirmed that HFD caused significant pathological changes extending fatty degeneration, karyomegaly, necrosis, and congestion (Figure 6(a)). These results are consistent with increased AST and ALT levels (Figures 6(d) and 6(e)) compared to the standard chow group. No treatment significantly altered these parameters after 14 doses (Figures 6(a), 6(d), and 6(e)). We also analyzed the kidney and pancreas infiltration of lymphocytes and plasmacytes (Figures 6(b) and 6(c)). HFD increased the number of these cells in the tissues, but no treatment significantly modified these numbers.

## 4. Discussion

In this study, we assessed the effects of PTAs from *Cecropia angustifolia* on obesity-associated metabolic disorders, using both cellular and diet-induced obesity mouse models to characterize their therapeutic potential impact on metabolic pathways linked to insulin resistance, dyslipidemia, and glucose homeostasis.

The connection between insulin resistance, T2DM, and dyslipidemia with obesity is well-documented [20]. Previous studies have shown that C57BL/6J mice on a HFD exhibit increased body weight, glucose intolerance, hyperinsulinemia, and hypercholesterolemia [21]. Our findings corroborate this, as the HFD-fed mouse model displayed similar metabolic alterations (Figures 3, 4, and 5).

The phytochemical profile of *Cecropia angustifolia* has identified compounds, such as PTAs, that potentially target key metabolic pathways, including those involved in glucose, insulin, lipid metabolism, and adiposity [22, 23]. Contrary to studies demonstrating weight-reducing effects from pentacyclic triterpenes [24], our study observed no significant weight changes in mice treated with PTAs, regardless of formulation or administration route (Figures 3(b) and 3(c)). The absence of this effect may be attributed to differences in extract composition or dosage. In our study, the PTA dosage was one-third of the amount used in studies where significant weight reduction was observed, which may explain the discrepancy [25]. In cellular assays with murine adipocytes, however, PTAs reduced TG accumulation, a finding consistent with similar studies in this cellular model [26, 27]. Notably, PTAs seemed to prevent further weight gain in mice, associated with an improvement in carbohydrate metabolism alterations, particularly when delivered intraperitoneally or orally in SIO, though the oral route was less effective, likely due to limited bioavailability caused by strong hydrophobicity. Interestingly, SIO had comparable effects, underscoring its role in glucose modulation independently of PTAs.

While PTAs did not influence plasma TGs, TC, or LDL cholesterol (Figures 5(b), 5(c), and 5(d)), their beneficial effects on carbohydrate metabolism were apparent. All treatments improved glucose tolerance and insulin sensitivity, as indicated by reductions in the AUC values for IPGTT and ITT compared to the HFD group (Figure 4). These results align with established triterpene activities, consistent with previous findings on PTAs' role in glucose regulation [24]. The route of administration influenced efficacy, with oral PTAs exhibiting weaker effects, possibly reflecting limited gastrointestinal absorption and bioavailability. Additionally, PTAs exhibited anti-inflammatory properties by reducing proinflammatory gene expression such as IL-1β (Figure 2(c)), aligning with known antioxidant and anti-inflammatory actions of pentacyclic triterpenoids [28, 29]. Toxicity evaluation demonstrated no adverse effects on liver, kidney, or pancreatic tissues, indicating that PTAs, irrespective of administration route, were nontoxic under study conditions (Figure 6).

An unexpected finding was that SIO significantly lowered blood glucose, and PTAs did not enhance this effect. This observation may be due to the high content of polyunsaturated fatty acids (PUFAs) in SIO, particularly alpha-linolenic and linoleic acids, known for their nutritional and antioxidant properties or its unsaponifiable fraction with hydrocarbons (squalene), waxes, sterols, alcohols, and tocopherols/tocotrienols [30, 31]. Previous studies in diabetic animal models similarly demonstrated that SIO improves glucose tolerance without affecting body weight or fat accumulation [32, 33]. Further analysis is needed to elucidate the mechanisms that produce these effects. No toxicity was observed in tissues of obese mice treated with SIO, further supporting its safety profile in chronic administration.

Overall, PTAs from *Cecropia angustifolia* modulated carbohydrate metabolism, showing hypoglycemic and anti-inflammatory activities without significantly altering lipid metabolism or body weight. SIO demonstrated comparable effects, suggesting that, at the administered dose, the combined PTAs and oil treatment did not produce additive or synergistic benefits. Future research should explore the mechanistic complexities of PTAs and further evaluate the potential of SIO as a functional component for metabolic health.

## 5. Conclusion

This study demonstrates the potential of PTAs from *Cecropia angustifolia* and SIO in addressing metabolic dysfunctions associated with obesity. PTAs, IP administered, significantly improved glucose tolerance and insulin sensitivity, exhibited anti-inflammatory properties, and prevented further weight gain without significantly altering lipid profiles or body weight. SIO also showed comparable hypoglycemic and anti-inflammatory effects, underscoring its standalone potential as a functional food ingredient. The lack of synergistic benefits when combining PTAs with SIO highlights the need for further research to explore their independent and combined mechanisms of action. These findings position PTAs and SIO as promising bioactive agents for managing metabolic health, demanding continued investigation into their therapeutic potential and optimal application strategies.

## Data Availability

The authors declare that the data supporting the findings of this study are available within the paper. Should any raw data file be needed in another format, they are available from the corresponding authors upon reasonable request.
